# Extensor Tenosynovitis due to *Mycobacterium marseillense* Infection in a Renal Transplant Recipient

**DOI:** 10.5435/JAAOSGlobal-D-20-00047

**Published:** 2021-01-12

**Authors:** Takashi Hirase, Jessica T. Le, Robert A. Jack, Todd E. Siff, Shari R. Liberman

**Affiliations:** From the Houston Methodist Orthopedic and Sports Medicine, Houston, TX.

## Abstract

Renal transplant recipients are at an increased risk of atypical nontuberculous mycobacterial (NTM) infections. Infections caused by NTM are uncommon in the general population, rarely occurring in immunocompetent individuals. NTM infections are an uncommon cause of tenosynovitis. *Mycobacterium marseillense* is a rare, atypical mycobacteria that has been reported to cause pulmonary and cutaneous infections; however, no previous reports of this pathogen causing tenosynovitis exist. This case reports a 73-year-old male renal transplant recipient who presented with chronic extensor tenosynovitis of the right hand caused by *M marseillense.* The patient was treated with radical extensor tenosynovectomy and 6 months of antibiotic treatment. A review of literature on tenosynovitis caused by atypical mycobacteria was performed. The patient successfully responded to treatment with no complications or recurrence of infection at the 18-month follow-up. Tenosynovitis of the hand caused by atypical mycobacteria is rare. A high index of suspicion is required to prevent a delay in diagnosis, particularly in immunocompromised individuals.

Immunocompromised patients including renal transplant recipients are at an increased risk of atypical nontuberculous mycobacterial (NTM) infections. Nontuberculous mycobacteria (NTM) are omnipresent environmental organisms, consisting of over 150 distinct species, found in soil, water, and other reservoirs.^1^ Infections caused by NTM are uncommon in the general population, rarely occurring in immunocompetent individuals.^2,3^ Manifestations of NTM infections can be observed as cutaneous disease, osteoarticular disease, pulmonary disease, or disseminated disease. The most observed species causing NTM infections in renal transplant recipients are *Mycobacterium chelonae* and *Mycobacterium kansasii*.^2,4^ Recently, *Mycobacterium marseillense* has been reported as the cause of pulmonary and cutaneous infections; however, there have been no previous reports of this pathogen causing tenosynovitis.

## Case Report

**Figure 1:**
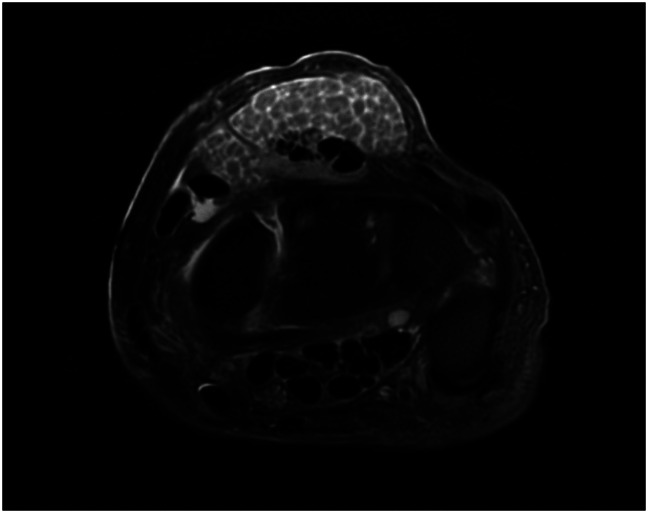
T2-weighted axial MRI of the right wrist demonstrate innumerable rice bodies within the fourth dorsal compartment with distension of the tendon sheath and a partial rupture of the extensor digitorum communis.

This case reports a 73-year-old man with a history of polycystic kidney disease after deceased donor kidney transplantation on tacrolimus, mycophenolic acid, and prednisone who presented to the office with a 1-year history of a progressively enlarging painless right dorsal wrist mass. He first noticed the mass a few weeks after his kidney transplant. He denied pain, weakness, or paresthesia in his right hand and wrist. His primary concern included the dorsal, painless mass. Examination revealed a soft-tissue mass 5 × 7 cm in size overlying the fourth dorsal compartment. The mass was soft, mobile, and nonpulsatile. The mass moved with flexion and extension of the digits. No neurovascular deficits were appreciated. MRI of the wrist demonstrated innumerable rice bodies within the fourth dorsal compartment with distension of the tendon sheath and a partial rupture of the extensor digitorum communis (EDC) tendon to the index finger (Figure 1). There was no preoperative detectable weakness in extension of the index finger. These findings along with a history of immunosuppression were strongly suggestive of infectious extensor tenosynovitis. Surgical intervention consisting of right dorsal wrist irrigation and débridement and extensor tenosynovectomy of the fourth dorsal compartment was proposed, and the patient elected to proceed.

A fourth dorsal compartment tenosynovectomy was performed, revealing innumerable tan-yellow rice bodies. The rice bodies were sent for culture and pathology. A partial rupture of the EDC tendon to the index finger (>50% intact) as previously seen on the MRI was identified and was treated with débridement alone. He tolerated the procedure well with no immediate complications and was discharged on the same day.

### Histopathologic Findings/Cultures

Histological examination of the specimen revealed synovial hyperplasia and numerous palisading granulomas with focal necrosis. Grocott methenamine silver and acid-fast bacilli (AFB) stains were performed and were negative for microorganisms. This pattern was suggestive of necrotizing granulomatous tenosynovitis. AFB culture yielded *M marseillense* after 10 days with susceptibility to all tested antibiotics (amikacin, linezolid, clarithromycin, and moxifloxacin). Aerobic, anaerobic, and fungal cultures yielded no growth after 3 days per our institutional protocol.

### Follow-up

**Figure 2:**
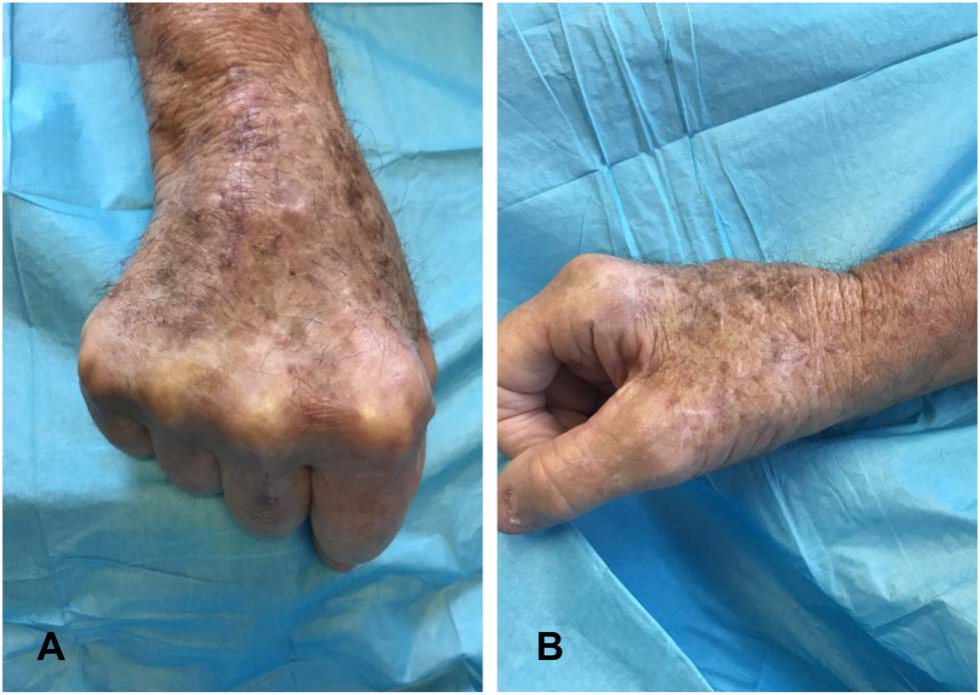
Photograph showing the dorsal (**A**) and lateral (**B**) aspects of the right wrist 3 months after radical extensor tenosynovectomy.

Because the culture yielded *M marseillense*, the infectious disease service recommended treatment with azithromycin 500 mg daily, rifabutin 300 mg daily, and ethambutol 400 mg daily to be taken for 6 months. At the 3-, 6-, 10-, and 18-month follow-ups, the incision was well-healed with no recurrence of mass (Figure 2). He tolerated the antibiotic treatment regimen well. His EDC function remained intact and had no neurovascular deficits.

## Discussion

To our knowledge, this is the first reported case of tenosynovitis caused by *M marseillense*. Among renal transplant patients, the reported incidence of NTM infections is between 0.16% and 0.38%.^5^ Atypical NTM infections in immunocompetent patients are uncommon, and when they occur, 90% of cases commonly affect the lungs, making NTM tenosynovitis an even rarer occurrence.^6^ These infections often occur because of previous trauma, surgery, corticosteroid injections, or nonapparent inoculation from contaminated sources. The most commonly reported site for NTM tenosynovitis is the fourth dorsal extensor compartment because of the highest risk of penetrating injury to this area in the hand and wrist.^6-8^ Previous case reports demonstrate favorable outcomes with no recurrence with adequate débridement and tenosynovectomy.^6-8^ In the case of our patient, no report of previous trauma or penetrating injury to the hand as a potential source for inoculation was observed. A variety of fast- and slow-growing NTM species have been isolated as causes of tenosynovitis, including *M chelonae*, *Mycobacterium malmoense*, *Mycobacterium abscessus* and more commonly, *Mycobacterium marinum* and *M kansasii*.^6-12^ *M marseillense* is a more recent discovery that until now has been described as causing infections with pulmonary, lymphatic, and cutaneous involvement.^3,13-15,21^

*Mycobacterium marseillense* was first described in 2009 as a new species belonging to the *Mycobacterium avium* complex phylogenetic group, originally isolated from respiratory specimens. It is a small, slow-growing, acid-fast, gram-positive bacilli showing close similarity to *Mycobacterium chimaera* and *Mycobacterium intracellulare*.^3,9,13,16^ Although NTM infections are generally uncommon in immunocompetent patients, there have been multiple reported cases of *M marseillense* occurring in immunocompromised patients. Therefore, one should include *M marseillense* in the differential diagnosis in treating individuals presenting with a slowly growing mass in the hand of an immunocompromised patient.

Clinical awareness of this entity is essential for diagnosis of atypical NTM tenosynovitis. Because *M marseillense* is slow-growing and painless, the time from onset to diagnosis can be anywhere from 6 months to 1 year. This delay in diagnosis and treatment can ultimately lead to tendinous disruption and bony involvement resulting in other complications. As noted in our patient, a partial tear of the EDC to the index finger was reported on the MRI and confirmed intraoperatively. The absence of clinical signs such as pain, fever, and erythema along with negative inflammatory markers such as a normal erythrocyte sedimentation rate and C-reactive protein make the diagnosis of *M marseillense* challenging.^12,17^ MRI can be helpful for diagnosis as well, showing synovial thickening around the tendons and fluid within the tendon sheaths. Rice bodies, seen on MRI or on gross intraoperative inspection may be suggestive but not pathognomonic for *M marseillense* as rice bodies can also be seen in other inflammatory conditions such as rheumatoid arthritis and seronegative inflammatory arthritis.^12,17,18^ Perioperatively, AFB cultures are essential for the diagnosis of *M marseillense*. Although AFB stains may be useful for immediate diagnosis of an NTM infection with >95% positive predictive value, it has been shown to have a 20% to 90% sensitivity depending on mycobacterial burden.^19^ Thus, current guidelines recommend continuing AFB cultures for 18 to 24 days to confirm negative growth after a negative AFB stain.^20^ Based on this case report, the authors propose consideration for tenosynovectomy and multidrug antimycobaterial therapy in the treatment of *M marseillense* in the hand and wrist.

## Conclusion

Tenosynovitis of the hand caused by atypical mycobacteria is rare. To our knowledge, this is the first reported case of *M marseillense* infection causing tenosynovitis in an immunocompromised patient. Diagnosis of tenosynovitis can be difficult because often no physical signs of acute or chronic infections exist, and erythrocyte sedimentation rate and C-reactive protein levels are typically normal. A high index of suspicion is required to prevent a delay in diagnosis and treatment, particularly in immunocompromised individuals.
